# The risk factors of bleeding after EUS-guided transmural drainage of pancreatic fluid collections: a single-center experience in China

**DOI:** 10.3389/fmed.2025.1626767

**Published:** 2025-07-30

**Authors:** Yaoting Li, Tingting Yu, Wei Zhang, Haiming Du, Yankun Hou, Jiao Tian, Senlin Hou, Lichao Zhang

**Affiliations:** Department of Biliopancreatic Endoscopic Surgery, The Second Hospital of Hebei Medical University, Shijiazhuang, Hebei, China

**Keywords:** EUS-guided drainage, peripancreatic fluid collections, bleeding, risk factors, single-center

## Abstract

**Background:**

Endoscopic ultrasonography (EUS) guided transmural drainage has become a first-line treatment for peripancreatic fluid collections (PFCs). Post-procedure bleeding may lead to severe clinical outcomes.

**Aim:**

The purpose of this study was to explore the patient-related and surgery-related factors associated with post-EUS drainage bleeding.

**Methods:**

This is an observational cohort study. A total of 181 patients who underwent EUS drainage at our center between June 2019 and May 2024 were enrolled analyzed in the study. Postoperative bleeding complications were observed, and patient and operation-related data were collected. Univariate and multifactorial logistics regression were performed for the risk factors that may affect postoperative bleeding. Determine the risk factors influencing bleeding after EUS drainage.

**Results:**

We achieved a 100% technical success rate. A total of 14 cases (7.7%) of bleeding occurred. All bleeding patients were successfully treated by conservative, endoscopic, interventional and other treatments. Logistic regression analysis showed that cyst size was an independent risk factor for bleeding after EUS-guided transmural drainage (*P* = 0.006; OR, 2.722; 95%CI, 1.327–5.587).

**Conclusion:**

The cyst size was an independent risk factor for bleeding after PFC drainage. Slowing the rate of decline in intracystic pressure may reduce the risk of bleeding.

## Introduction

Peripancreatic fluid collections (PFC) is usually secondary to acute pancreatitis, chronic pancreatitis and pancreatic trauma. Treatment methods include surgery, percutaneous intervention, and endoscopic drainage. Multiple studies have demonstrated that endoscopic drainage is safe and effective (1, 2).

Endoscopic drainage of PFCs was first reported in the late 1980s (1). After nearly 40 years of development, this technique has become the first-line treatment for acute peripancreatic effusion (2–4). Bleeding after EUS drainage is a rare adverse event with an incidence of about 2–10%, and the incidence varies widely among centers due to sample size limitations (2, 4). At present, there are few studies on bleeding after EUS drainage, and only a few case reports have reported endoscopic management of bleeding. Mild bleeding can be treated conservatively, and severe bleeding requires re-interventional treatment or even surgery. This increases the length and cost of hospitalization and affects patient recovery. Therefore, it is very important to find the risk factors of bleeding after endoscopic drainage of PFCs. In this study, we aimed to determine the related risk factors of bleeding after EUS drainage.

## Materials and methods

### Patients

We analyzed patients who underwent EUS-guided transmural drainage in our center from June 2019 to May 2023. All patients with symptomatic PFC were collected and analyzed in the study. The inclusion criteria were: 1. Symptomatic PFC mainly includes abdominal distension, abdominal pain, nausea, jaundice, etc.; 2. PFC that was resistant to conservative treatment; 3. All patients had a disease course of more than 4 weeks; 4. There is no strict age limit. Patients under the age of 18 must obtain informed consent from their guardians. The exclusion criteria were: 1. Enhanced CT observed a patient with a high density in the cyst cavity suspected of hemorrhage; 3. Suspicion of malignancy. All patients with platelet abnormalities, coagulation abnormalities, and conditions with cirrhosis or esophageal varices that could affect postoperative bleeding were excluded from the study. A total of 181 patients were finally enrolled in the study.

### Ethics

This was a retrospective study in which we obtained informed consent and signature from patients before collecting their data. The study was approved by the Ethics Committee of our center (2021-R158). I confirm that all methods are carried out in accordance with the relevant guidelines and regulations

### Variables

Potential risk factors that may affect postoperative bleeding were collected as follows. The factors related to patients included gender, age, etiology, cyst diameter, cyst type, and cyst wall thickness. The factors associated with EUS drainage mainly included postoperative infection and stent type, location of puncture, Number of puncture channels. Firstly, univariate regression analysis of risk factors was performed, and related factors with *P* < 0.05 were included in multivariate regression analysis, and independent risk factors affecting bleeding were identified.

### Procedure

**FIGURE 1:**
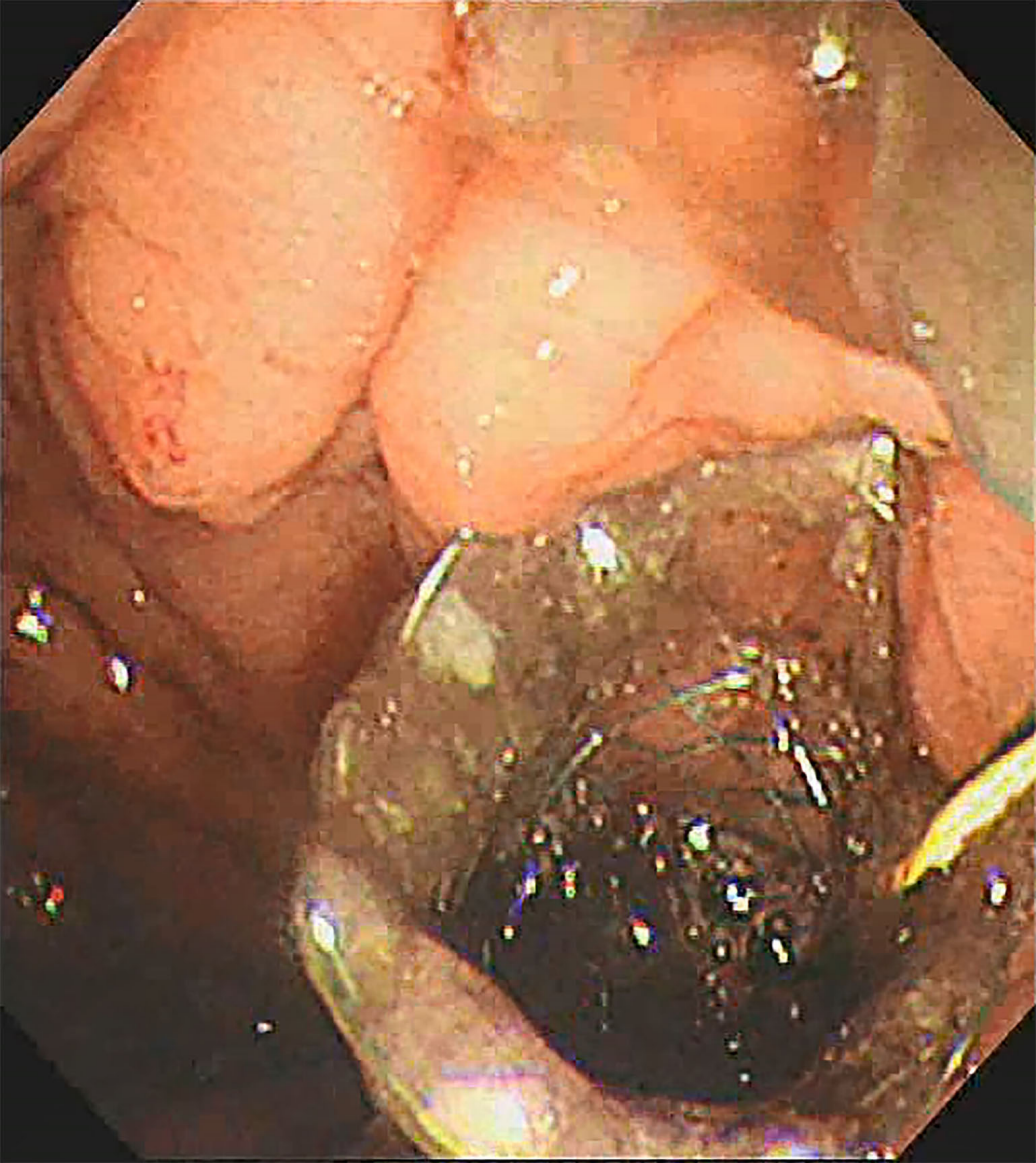
The cyst was accurately scanned by EUS.

**FIGURE 2:**
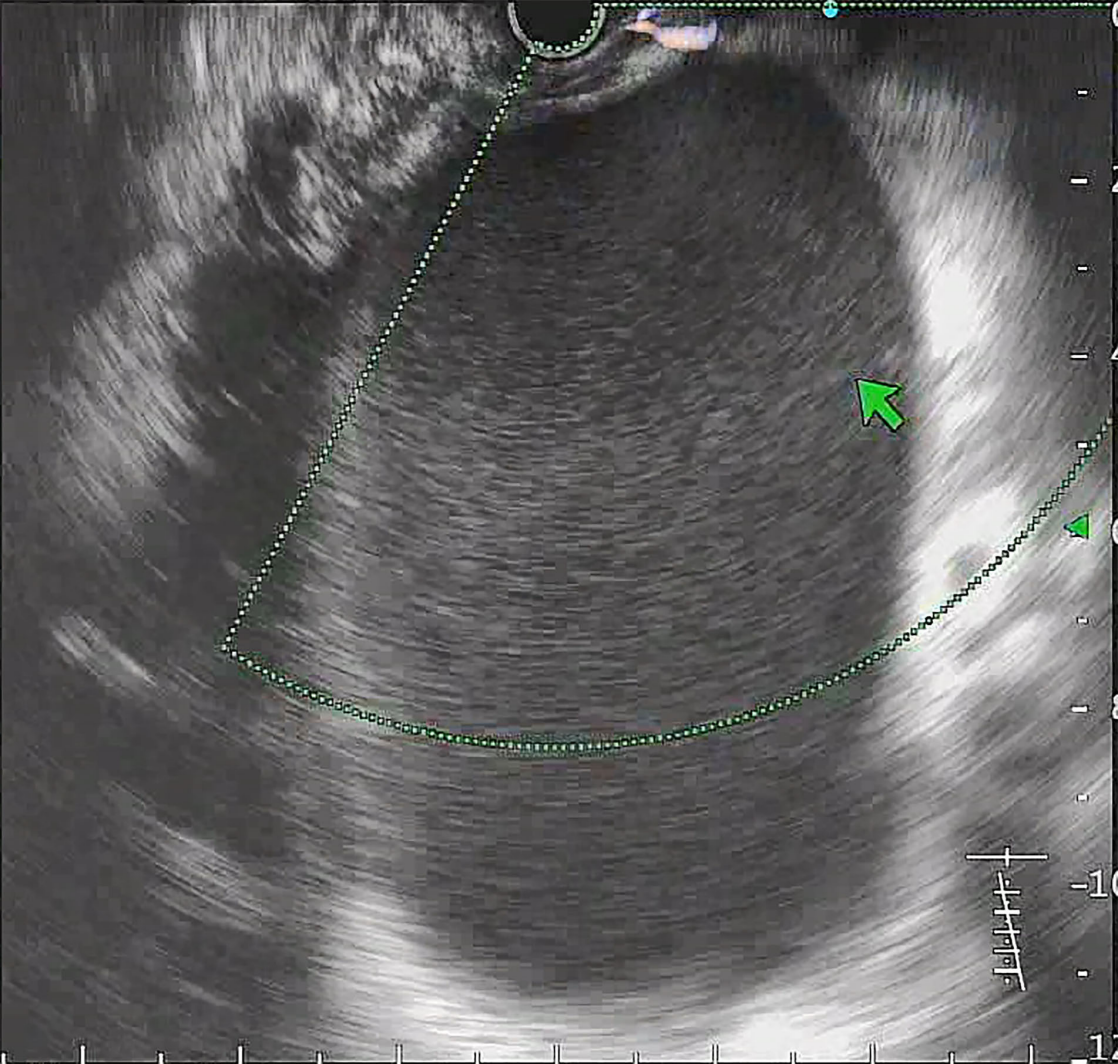
The double pigtail plastic stent was inserted into the cyst through the guide wire.

**FIGURE 3:**
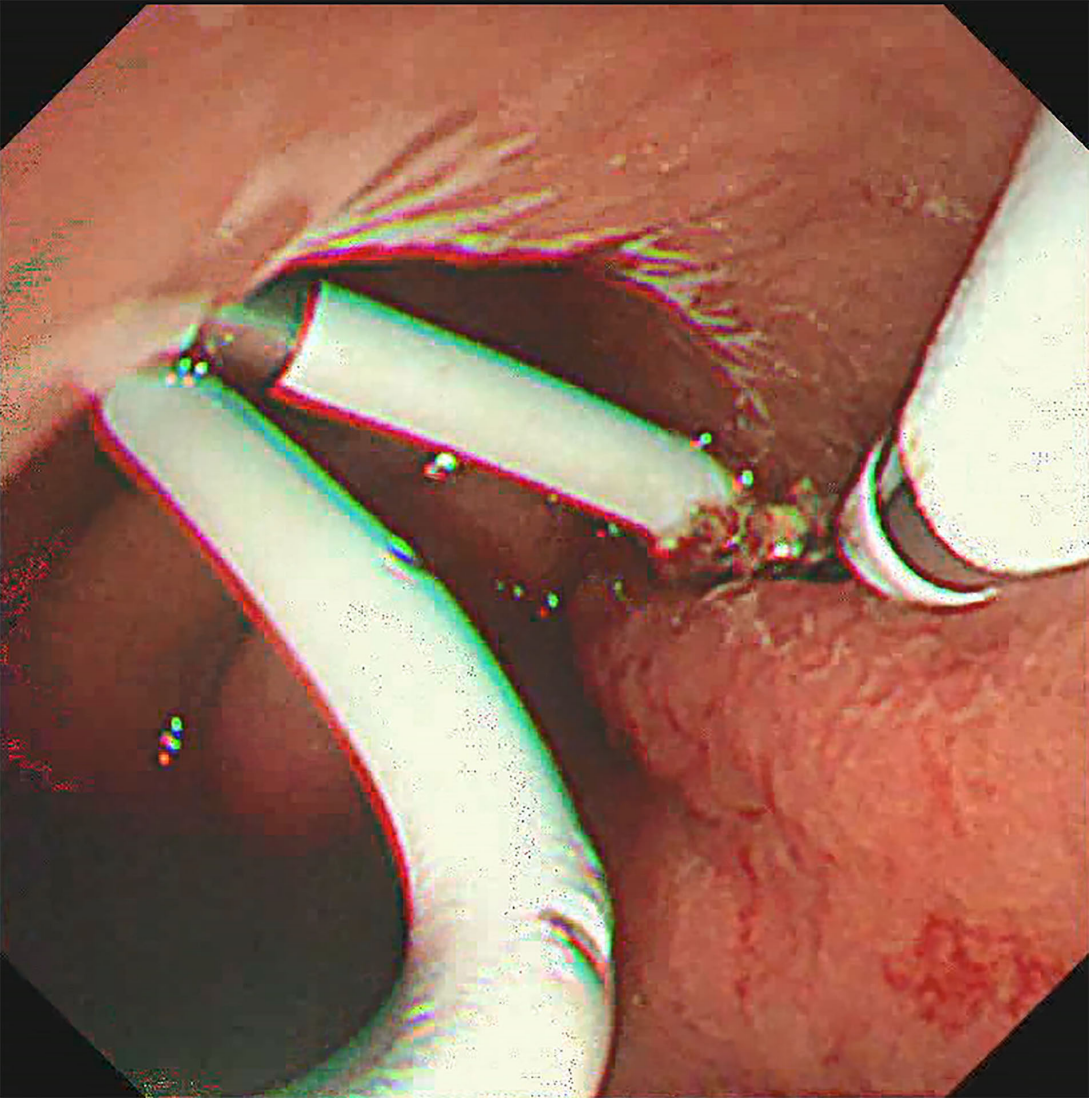
The plastic metal stent was placed into the cyst through the guide wire.

All procedures were performed by experienced endoscopists under intravenous anesthesia. Endoscopic ultrasound (ME2, OLYMPUS, JAPAN) was used to perform standard scanning of the pancreas (shown in Figure 1), and bleeding and solid nodules will be excluded if detected. The cyst was then punctured with puncture needle (ECHO-19, COOK, United States) at a suitable location (stomach or duodenum) avoiding blood vessels, A 0.035-inch guidewire (Jagwire, Boston Scientific, United States) was inserted through the needle lumen into the cyst. A cystotome (10 Fr, COOK, United States) was used to dilate the puncture tract. Plastic or self-expanding metal stent (SEMS Boston Scientific, United States) and nasal cyst tubes (for postoperative irrigation) were then placed depending on the size of the cyst and inside necrosis (shown in Figures 2, 3). All patients with metal stents were fitted with fully coated self-expanding metal stents, and all patients with plastic stents were fitted with two 7Fr diameter double pigtail plastic stents. Nasal cyst tubes were routinely inserted in all patients to facilitate postoperative observation of complications such as bleeding. To prevent infection, routine prophylactic treatment with antibiotics (ceftriaxone, 2g, once daily, intravenously) was given 3 days before surgery. Abdominal CT was reexamined at 7 and 30 days after surgery, and then once a month. The stent was removed when the patient’s symptoms had disappeared for at least 2 weeks and CT confirmed the cyst had disappeared.

### Definition

Technical success was defined as access to the PFC by placement of a stent and drainage. Clinical success is defined as the disappearance of symptoms, a reduction in PPC size (more than 50%), or complete regression (2). If the fever exceeds 38 degrees Celsius for more than 24 h after EUS drainage, it is defined as an infection. After the operation, rinse with the nasal cyst tube. A decrease of 2 g/L in hemoglobin after surgery, or the occurrence of hematemesis and melena, is defined as bleeding (5). The classification of adverse events is divided into six levels. A:No treatment is needed; B: have minor therapy or consequence, including overnight admission; C: that requires major therapy or hospitalization (24–48 h); D:major therapy, need unplanned increase in level of care, or hospitalization > 48 h; E:result in permanent adverse sequelae; F: death (5). Re-intervention was defined as the need for repeat surgery or endoscopy owing to persistent symptoms in association with a residual pseudocyst that was not less than 50% of the original size on follow-up imaging (6). All patients were divided into two groups according to the QNI system: the group with lower QNI stratification (≤ 2 quadrants and necrosis rate ≤ 30%) and the group with higher stratification (≥ 3 quadrants, necrosis rate ≥ 30% in 2 quadrants or necrosis and infection rate > 60% in 1 quadrant) (7). After the occurrence of bleeding symptoms, red blood cells were transfused in time, hemostatic drugs were applied, and endoscopic hemostasis or vascular interventional embolization was performed if necessary. If infection or bleeding is difficult to control, embolismor surgical intervention may be considered. The type of cyst was defined by the amount of solid necrotic material in the cyst. The procedure was performed by experienced endoscopists and imaging specialists. The size of the cyst and necrotic material was measured by ultrasound images and nuclear magnetic images. According to Atlanta classification (8), if the solid debris in the cyst was greater than 50%, it was identified as walled off necrosis (WON), and if it was less than 50%, it was identified as pancreatic pseudocyst (PPC).

### Statistical analysis

The sample size of our study is estimated based on the number of independent variables (greater than 15 times the number of independent variables). Data were analyzed using SPSS 27.0 (SPSS Inc., Chicago, Illinois, United States). Results are expressed as medians and means and ranges. A *P*-value below 0.05 was considered statistically significant. Univariate analysis for screening purposes for risk factors. In addition, multivariate analysis calculating the hazard ratio using logistic binary regression was added on the factors identified by univariate analysis as independently significant for bleeding after EUS—guided transmural drainage.

## Results

### Patient and PFC characteristics

A total of 181 patients were enrolled in the study, including 111 male and 70 female with a mean age of 49 years (range 8–85 years). Their baseline characteristics were shown in Table 1. The causes of PFC were acute pancreatitis in 150 cases (82.9%), chronic pancreatitis in 17 cases (26%), surgery in 6 cases (3%) and trauma in 8 cases (4%). The median diameter of the cyst was 10.9 cm (range 4.1–25.3 cm). Among these patients, 21 (11.6%) developed symptoms of infection (fever or elevated WBC count). In our process, 179 cases were punctured through the stomach and 2 cases were punctured through the duodenum. The average thickness of the cyst wall was 4.6 mm (1—10 mm). 161 patients were identified as PPC and 20 patients were identified as WON. Five patients were treated with dual-channel puncture (puncture at two locations with stent or nasal cyst tube inserted) and 176 patients were treated with single-channel puncture (puncture at only one location with stent or nasal cyst tube inserted).

**TABLE 1:** Baseline characteristics of patients who underwent EUS-guided drainage.

The total number of patients	181
**Gender**	
Male	111(61.3%)
Female	70(38.7%)
Age (year)	49
**Etiology**	
Acute pancreatitis	150(82.9%)
Chronic pancreatitis	17(26%)
Surgery	6(3%)
Trauma	8(4%)
Cyst diameter (cm)	10.9
**Location of cyst**	
Head and neck	10(5.5%)
Body and tail	171(94.5%)
**Type of cyst**	
PPC	161(89%)
WON	20(11%)
Cyst wall thickness (mm)	4.6
**Infection (Before operation)**	
Yes	21(11.6%)
No	160(88.4%)
**Puncture channel**	
Single channel	176 (97.2%)
Dual channel	5 (2.8%)
**Type of stent**	
Metal stent	12 (6.6%)
Plastic stent	169 (93.4%)

### Technical outcomes

All patients were successfully drained by EUS and the stent (plastic or metal) was successfully removed after the cyst was identified and symptoms resolved postoperatively. The median follow-up period was 11.5 months, and 9 (4.9%) patients recurred, 7 patients were managed by re-endoscopic intervention, and 2 patient was managed by surgery. In our cohort, 22.6% of patients developed symptoms of infection. Among them, 4 patients underwent repeated endoscopic removal of necrotic tissue, the remaining 38 patients with symptoms of infection disappeared after antibiotics and nasal cyst tube irrigation. Among the classification of adverse infection events, 2 patients were classified as grade C and 40 patients as grade D. Among these patients, 14 (7.7%) had bleeding after EUS drainage. Seven patients were treated with conservative therapy for hemostasis, two patients were treated with endoscopic self-expandable partially covered metal stent for hemostasis, three patients were treated with endoscopic hemostasis clamp, and two patients was treated with vascular embolism for hemostasis. All bleeding patients were infused with suspended red blood cells and rehydration to improve clinical symptoms. All patients with bleeding were classified as grade D according to the classification of adverse events. No serious adverse events such as piercings and death occurred.

**FIGURE 4:**
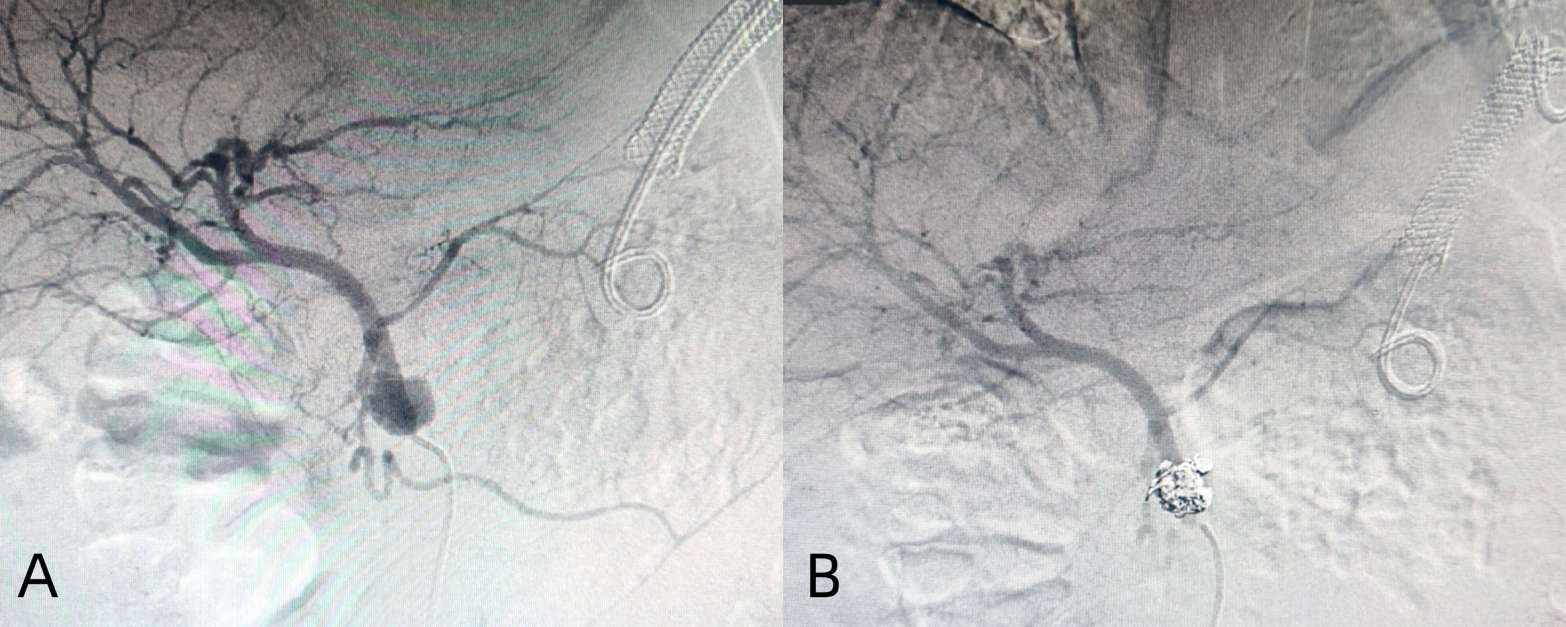
Angiography revealed contrast agent overflow **(A)** and hemostasis was performed **(B)**.

**FIGURE 5:**
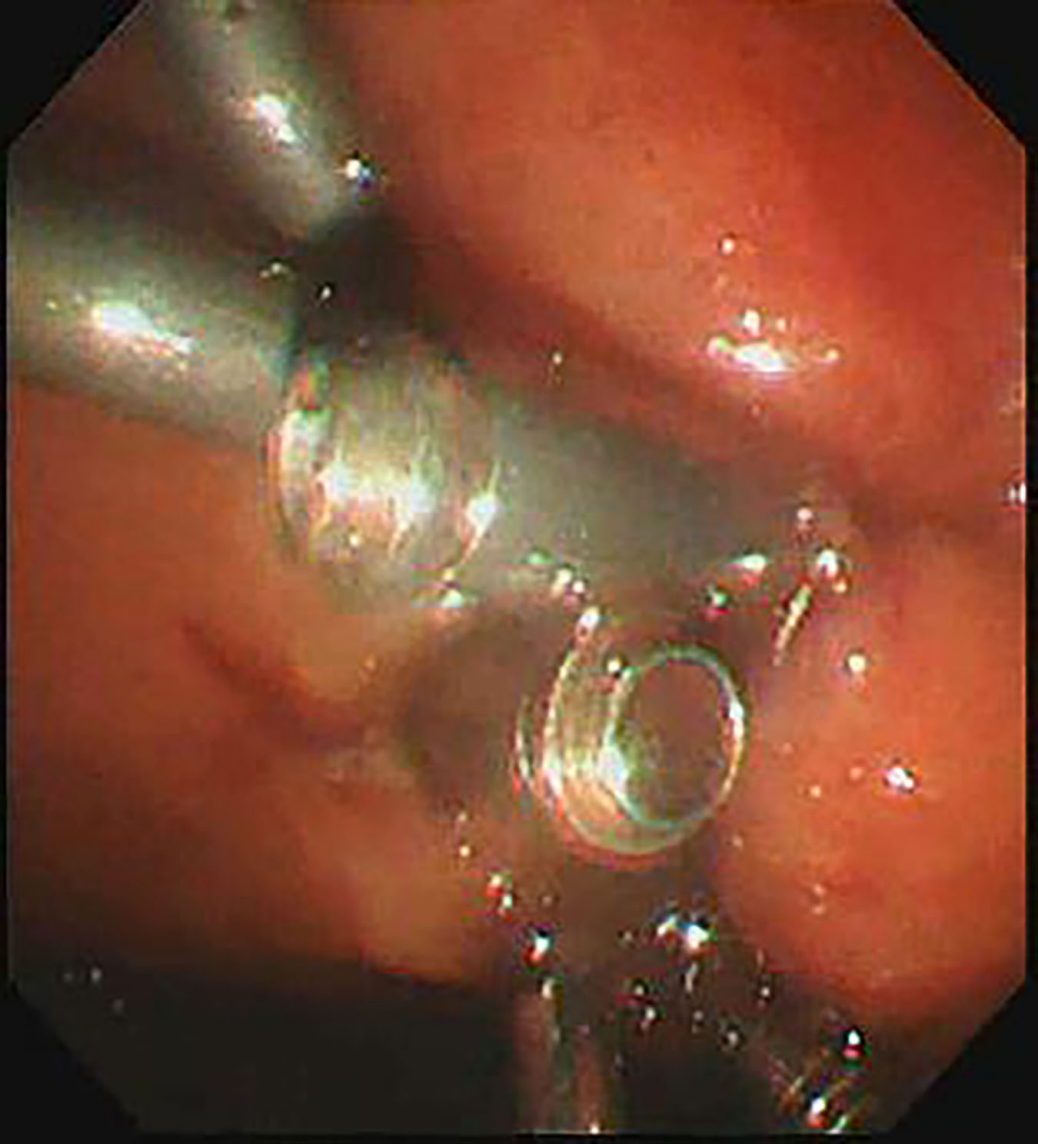
Hemostasis was achieved by clamping with a hemostatic clamp.

The treatment methods of endoscope hemostasis include metal stent placement, hemostatic clamp clamping, balloon packing and compression. In our patients with hemorrhage, two cases were caused by vascular variation in the cyst, and the hemostasis was successfully stopped by vascular interventional embolization (Figure 4). There were 5 patients with bleeding at the puncture site and through the puncture channel, and the bleeding was successfully stopped by the implantation of the self-expandable partially covered metal stent and application of the hemostatic clamp (Figure 5). In seven patients, the cause of bleeding was unclear, but hemostasis was eventually achieved through conservative treatment. All bleeding occurred 24 h after the operation, the minimum time was 24 h, and no bleeding occurred during the operation. The longest bleeding time was 7 days after surgery. Tailed characteristics of all bleeding patients are shown in Table 2.

**TABLE 2:** Detailed characteristics of all bleeding patients.

Patient number	Cyst type	Type of stent	Bleeding time (after operation time)	Cause of bleeding	Treatment measure
1	Pancreatic pseudocyst	Plastic stent	1d	unknown	Conservative treatment
2	Pancreatic pseudocyst	Plastic stent	1d	Puncture channel bleeding	covered metal stent was placed in the puncture channel
3	Pancreatic pseudocyst	Plastic stent	2d	Blood vessel bleeding in cyst	Vascular intervention
4	Walled off necrosis	Plastic stent	4d	Puncture point bleeding	Titanium clamp to stop bleeding
5	Pancreatic pseudocyst	Plastic stent	7d	unknown	Conservative treatment
6	Pancreatic pseudocyst	Plastic stent	6d	Puncture point bleeding	Titanium clamp to stop bleeding
7	Walled off necrosis	Plastic stent	3d	unknown	Conservative treatment
8	Walled off necrosis	Plastic stent	4d	Puncture channel bleeding	covered metal stent was placed in the puncture channel
9	Pancreatic pseudocyst	Plastic stent	5d	Blood vessel bleeding in cyst	Vascular intervention
10	Walled off necrosis	Metal stent	3d	unknown	Conservative treatment
11	Pancreatic pseudocyst	Plastic stent	3d	unknown	Conservative treatment
12	Pancreatic pseudocyst	Plastic stent	1d	Puncture point bleeding	Titanium clamp to stop bleeding
13	Pancreatic pseudocyst	Plastic stent	2d	unknown	Conservative treatment
14	Pancreatic pseudocyst	Plastic stent	1d	unknown	Conservative treatment

### Risk factors of bleeding after EUS drainage

Univariate and multivariate analyses were performed for possible factors related to bleeding after EUS-guided transmural drainage, and Univariate analysis (shown in Table 3) showed that cyst type and cyst size were risk factors for bleeding after EUS guided transmural drainage (*P* = 0.04 and *P* = 0.004). Multivariate analysis (shown in Table 4) showed that the cyst size was statistically significant (*P* = 0.006, *OR* = 2.722). Cyst size influence bleeding after EUS transmural drainage, and cyst size was an independent risk factor. The larger the cyst, the higher the risk of bleeding after endoscopic drainage.

**TABLE 3:** Univariate analysis of the risk factors for bleeding after EUS-guided drainage.

Variable	Bleeding after EUS drainage	*P*	OR
Gender		0.180	2.457
Male	11/111(10%)		
Female	3/70(4%)		
Age (year)		0.535	0.698
>50	9/102(8.8%)		
≤ 50	5/79(6%)		
Etiology		0.933	-
Acute pancreatitis	13/150(8.7%)		
Chronic pancreatitis	0/17(0%)		
Surgery	1/6(16.7%)		
Trauma	0/8(0%)		
Cyst diameter (cm)		0.004*	2.836
≤ 10	4/97(4.1%)		
>10 and ≤ 15	3/55(5.5%)		
>15	7/29(24.1%)		
Type of cyst		0.04*	0.265
PPC	10/161(6.2%)		
WON	4/20(2%)		
Cyst wall thickness (mm)		0.881	1.107
≤ 5	11/145(7.6%)		
>5	3/36(8.3%)		
Infection		0.745	0.770
Yes	2/21(9.5%)		
No	12/160(7.5%)		
Stent type		0.936	1.091
Metal	1/12(8.3%)		
Plastic	13/169(7.7%)		
Location of puncture		0.078	12.768
Transgastric	13/179(7.3%)		
Transduodenal	1/2(50%)		
Puncture channel		0.322	0.319
Single channel	12/176(6.8%)		
Dual channel	2/5(40%)		

*Statistically significant.

**TABLE 4:** Multivariate analysis of the risk factors for bleeding after EUS-guided drainage.

Variable	B	SE	Wals	df	P	OR	OR; 95%CI
Type of cyst	–1.193	0.679	3.091	1	0.079	0.303	0.08–1.147
Cyst diameter	1.002	0.367	7.456	1	0.006*	2.722	1.327–5.587

*Statistically significant.

According to the QNI system, 18 patients were assigned to the high QNI group and 163 patients were assigned to the low QNI group. There was no significant difference in the incidence of adverse events such as bleeding and infection between the two groups of patients (*P* > 0.05) (Table 5).

**TABLE 5:** Analysis of adverse events after EUS drainage in the QNI high group and the QNI low group.

Variable	High QNI group	Low QNI group	*P*
Infection	4/18(22.2%)	38/163(23.3%)	0.583
Bleeding	1/18(11.1%)	13/163(7.4%)	0.591

## Discussion

The clinical manifestations of PFC vary widely, from asymptomatic to fatal (9). In general, patients with cysts smaller than 5 cm in diameter or asymptomatic patients do not need intervention. Although there are many kinds of intervention methods, endoscopic EUS-guided transmural drainage has become the preferred treatment for PFC (6, 10, 11). In a prospective study, endoscopic drainage was associated with less cost and shorter hospital stays (6). Because there was no abdominal wall incision and no abdominal drainage tube, the acceptance level of patients is higher. Especially in the lead generation of WON, the advantages of EUS lead generation are even more obvious (12). Moreover, a number of studies have proved that EUS-guided transmural drainage was safe and effective (13, 14), which has also been confirmed in our center. Although EUS can accurately scan the blood flow of the patient’s stomach wall and cysts to avoid damaging them. However, postoperative bleeding also occur after endoscopic drainage. This can lead to increased hospital costs and prolonged hospital stays, and even potentially life-threatening adverse events. Therefore, we tried to find the risk factors causing bleeding after transmural drainage of PFC in the present study.

Previous studies have reported a bleeding rate of 2–10% in EUS-guided transmural drainage (2, 15). This is biased in different centers. In our cohort, the bleeding rate after EUS-guided transmural drainage was 7.7%, which was similar to that previously reported. In univariate analysis, the etiology of cyst formation, the thickness of cyst wall and the type of scaffold had no effect on bleeding after EUS drainage. The type and size of the cyst are risk factors for bleeding after transmural drainage.

The cause of bleeding after EUS-guided transmural drainage is unknown. Studies have suggested that pseudoaneurysm is an important cause of bleeding (16). But it seems to be limited to intra-procedural bleeding. There are related reviews that the main causes of post-procedural bleeding are coagulation disorders and stent type (16, 17). Initially, it was thought that patients with metal stents were more likely to bleed (16, 18). But this is controversial. Many recent studies have concluded that metal stents do not increase the risk of postoperative bleeding (19, 20). Meanwhile, some studies suggest that for the treatment of WON, the therapeutic effect of metal stents may be better (21). The stent type did not increase the risk of bleeding (*P* = 0.936) in our study. This might be because the types of metal scaffolds we adopted were all self-expanding metal scaffolds. Because in several studies on lumen-apposing metal stent (LAMS), the incidence of postoperative bleeding with LAMS stents was relatively high (22, 23). Both univariate and multivariate analysis showed that the size and type of cyst affected the bleeding after drainage. And cyst size was an independent risk factor (OR, 2.722; 95%CI, 1.327–5.587). For the cyst type, won increases the risk of postoperative bleeding, which may be due to chronic erosion of blood vessels due to excessive solid debris within won, leading to postoperative bleeding. In terms of cyst size, the larger the cyst, the higher the risk of bleeding after endoscopic drainage. In our study, the risk of bleeding increased significantly (24.1%) when the cyst was about 15 cm in diameter. This may be due to the pressure in the sac of the larger cyst dropping too fast, causing the blood vessels in the sac to dilate rapidly, leading to bleeding. But further studies are needed to confirm this.

Bleeding after EUS-guided transmural drainage can be treated by conservative therapy, endoscopic therapy, vascular intervention and surgery. But so far there was no consensus or guidelines, and only a few cases have been reported (16). Conservative treatment may be effective for patients with mild bleeding. For patients with severe bleeding, timely endoscopic hemostasis is necessary. In previously reported cases, endoscopic hemostasis has been remarkably effective (24–26). For the stomach wall, the duodenal wall may be weaker and have a richer blood supply. Therefore, both puncture location and number of puncture channels may affect bleeding, but in our cohort, neither of these factors affected postoperative bleeding. This may be due to the fact that very few of our patients have undergone transduodenal and double-channel puncture. As shown, large cysts and erosion of necrotic materials may be the main causes of postoperative bleeding. Proper identification of cyst types and accelerated necrotic, solid material expulsion may reduce the risk of postoperative bleeding. At the same time, for patients with larger cysts, slowing the rate of decline in intracystic pressure may reduce the risk of bleeding. Endoscopic management of bleeding after EUS-guided transmural drainage is a very challenging procedure, therefore, identification of risk factors and preventive strategy is important (16).

The study also had some limitations. First of all, the study was a single-center study, so we couldn’t get a larger sample size to include more risk factors. This may lead us to overlook some potential risk factors. For instance, in a recent predictive study on EUS metal stent drainage, patients with preoperative evidence of pancreatic duct leakage/rupture had a higher incidence of adverse events (27). Therefore, some prospective studies with large sample sizes are necessary. Overall, more research and standardized procedures for bleeding management are needed in the future.

## Conclusion

The cyst size was an independent risk factor for bleeding after PFC drainage. Slowing the rate of decline in intracystic pressure may reduce the risk of bleeding.

## Data Availability

The raw data supporting the conclusions of this article will be made available by the authors, without undue reservation.
